# Further Evidence That Science-Based Biosecurity Provides Sustainable Prevention of Porcine Reproductive and Respiratory Syndrome Virus Infection and Improved Productivity in Swine Breeding Herds

**DOI:** 10.3390/ani14172530

**Published:** 2024-08-30

**Authors:** Scott Dee, Lisa Brands, Roy Edler, Adam Schelkopf, Joel Nerem, Gordon Spronk, Mariana Kikuti, Cesar A. Corzo

**Affiliations:** 1 Pipestone Research, Pipestone, MN 56164, USA; roy.edler@pipestone.com; 2 Pipestone Veterinary Services, Pipestone, MN 56164, USA; lisa.brands@pipestone.com (L.B.); adam.schelkopf@pipestone.com (A.S.); joel.nerem@pipestone.com (J.N.); gordon.spronk@pipestone.com (G.S.); 3 College of Veterinary Medicine, University of Minnesota, St. Paul, MN 55108, USA; mkikuti@umn.edu (M.K.); corzo@umn.edu (C.A.C.)

**Keywords:** pigs, PRRS, virus, biosecurity, incidence, risk, key, performance, indicators, breeding herds, prevention

## Abstract

**Simple Summary:**

Porcine reproductive and respiratory syndrome virus (PRRSV) infection in sows results in reduced animal welfare, poor breeding herd performance, and economic loss to farmers. The best way to reduce the effects of PRRSV is to prevent its entry into swine breeding herds through improvements in farm biosecurity. To accomplish this goal, the concept of Next Generation Biosecurity (NGB) was developed. A recent publication demonstrated that the practice of a comprehensive, science-based program of biosecurity (NGB COMPLETE) which incorporated science-based biosecurity protocols targeting direct and indirect routes of PRRSV transmission, significantly reduced PRRSV incidence risk in breeding herds compared to a partial program (NGB INCOMPLETE). This new communication follows up on this earlier paper and brings new information by reporting significant differences in key performance indicators between NGB COMPLETE breeding herds versus NGB INCOMPLETE herds over that original 2-year period across the same swine production system. It also supplements the earlier paper with data from a third consecutive year of reduced PRRSV incidence risk, resulting in a PRRSV incidence risk of 8.0% across all breeding herds for the entire three years. In closing, this is further evidence demonstrating that NGB, while not perfect, brings value to farmers through sustainable prevention of PRRS and improved productivity.

**Abstract:**

Porcine reproductive and respiratory syndrome virus (PRRSV) is a globally significant pathogen of pigs. Preventing the entry of PRRSV into swine breeding herds enhances animal health and welfare. A recently published retrospective cohort study reported significant differences in PRRSV incidence risk between breeding herds that practiced Next Generation Biosecurity (NGB) COMPLETE, versus herds that practiced a partial approach (NGB INCOMPLETE) over a 2-year period. This follow-up communication builds on this previous publication and brings new information regarding statistical differences in key performance indicators (KPIs) from 43 NGB COMPLETE herds and 19 NGB INCOMPLETE herds during disease years 1 and 2. Statistically significant differences included higher total born/farrow and pigs weaned/female along with a reduced pre-weaning mortality and wean to 1st service interval, as well as a 0.91 increase in the number of pigs weaned/mated female/year. In addition, this communication reports that PRRSV incidence risk throughout disease years 1–3 was 8.0%, and that the association of NGB status (COMPLETE vs. INCOMPLETE) and disease burden for the cumulative 3-year period was statistically significant (*p* < 0.0001). These findings support previously published data that NGB, while not perfect, provides sustainable prevention of PRRSV, and may help improve herd productivity.

## 1. Introduction

Porcine reproductive and respiratory syndrome (PRRS), a globally significant disease of pigs, is caused by the porcine reproductive and respiratory syndrome virus (PRRSV) [1]. Infection of breeding female swine with PRRSV results in reduced animal welfare, poor reproductive performance, and significant economic loss to farmers [2]. To control PRRS, PRRSV entry into breeding herds must be prevented through the application of a comprehensive, science-based plan of biosecurity known as “Next Generation Biosecurity” (NGB) [3]. As previously described, NGB prevents viral entry through the application of specific routes of PRRSV transmission, including direct routes (infected pigs and contaminated semen) and indirect routes (mechanical/fomite-based routes), aerosols, and feed through the application of science-based biosecurity protocols [4,5,6,7,8]. For review, the protocols of biosecurity designed to prevent viral entry via these routes have been applied, tested, and published [3]. Specifically, NGB was applied to breeding herds managed by Pipestone Veterinary Services within a large-scale commercial swine production operation known as the Pipestone System, the third largest swine production company in the United States [3]. Historically, from July 2009 to June 2021, annual PRRSV incidence risk across Pipestone-managed breeding herds ranged from 15% to 55% (Table 1).

To drive change, NGB was initially applied for a 2-year test period (1 July 2021–30 June 2023), and PRRSV incidence risk was analyzed using a retrospective cohort design across 321,013 sows from 69 managed breeding herds (disease year 1) and 381,404 sows from 76 managed breeding herds (disease year 2) [3]. Over this time, the application of NGB reduced PRRSV incidence risk to 8.6% in disease year 1 and 9.2% in disease year 2 [3]. The analysis also identified two cohorts: one that implemented protocols for all phases of NGB (NGB COMPLETE, 56 herds) and those that implemented all phases of NGB biosecurity except air filtration (NGB INCOMPLETE, 20 herds). Over the two disease years, PRRSV incidence risk in NGB COMPLETE herds was 8.9% versus 40.0% in NGB INCOMPLETE herds, and the association between NGB status and PRRSV incidence risk for the 2-year period was statistically significant (*p* = 0.006), favoring the NGB COMPLETE approach [3]. This paper provided the initial evidence that improvements in biosecurity resulted in a sustainable reduction of PRRSV incidence risk in breeding herds in a large commercial swine production system. However, limitations of this report included no information on differences in key performance indicators (KPIs) during the 2-year assessment period between the two cohorts, or if a reduced level of PRRSV incidence risk could be sustained for a third consecutive year (disease year 3). Therefore, the purpose of this follow-up communication was to report PRRSV incidence risk for a disease year 3, to analyze the association between biosecurity status (NGB COMPLETE vs. NGB INCOMPLETE) and disease burden (PRRSV incidence risk) over the cumulative three-year period, and to statistically assess key performance indicators between NGB COMPLETE breeding herds and NGB INCOMPLETE herds in disease years 1 and 2.

## 2. Materials and Methods

### 2.1. Ethical Review

Following the Pipestone Research IACUC review, it was determined that an ethical review of the farms reported for the pending assessments was not needed, as the original study was data-based, and this new paper was a follow-up in support. In addition, Pipestone Veterinary Services has permission to use site data for research and publication as part of the management contracts.

### 2.2. Descriptive Data from Participating Herds in Disease Year 3

Table 2 describes data from breeding herds that participated in disease year 3. Included are data from the total number of participating herds, as well as the number of herds in each cohort, NGB COMPLETE and NGB INCOMPLETE.

### 2.3. Herd Selection and Statistical Analysis of KPIs across Cohorts

For assessment of KPIs, only herds that provided two complete years of data during disease year 1 (1 July 2021 to 30 June 2022) and disease year 2 (1 July 2022 to 30 June 2023) were selected. This dataset was controlled through the use of a standardized nutrition program (Pipestone Nutrition, Pipestone, MN, USA), and a farm management company practicing consistent employee training programs and animal handling protocols (Pipestone Management, Pipestone, MN, USA). This approach ensured that all farm workers were employees of the company, had been trained using consistent protocols, and underwent regular auditing and performance reviews, maximizing both stability in the labor force and compliance with biosecurity protocols. In addition, one record-keeping system (Porcitec, Agritecsoft.com, Barcelona, Spain) was used, and animal health programs and products were provided by one source (Pipestone Veterinary Services, Pipestone, MN, USA). To maximize sample size, all genetic sources (n = 3) employed within the company during the two years were included. This decision was justified using a generalized linear model which indicated no interaction between genetic source and treatment (NGB COMPLETE and NGB INCOMPLETE) across any of the KPIs (Edler R., Pipestone Research, Pipestone, MN, USA, personal communication, 31 July 2024). Following final selection, breeding herds were defined as either NGB COMPLETE, involving protocols to mitigate the defined designated direct and indirect routes, or NGB INCOMPLETE, protocols to mitigate direct and indirect routes in the absence of air filtration, as described [3]. Across qualifying herds, KPIs from the Porcitec performance analysis were analyzed. The KPIs evaluated included the following: farrowing rate (%); total number of pigs born/farrow; number of pigs weaned/female; % pre-weaning mortality; number of pigs weaned/mated female/year; % repeat services; average parity of sows farrowed; % multiple matings; wean to first service interval (days); % sows bred by 5 days; conception rate (%); farrowing rate (%); age at first service (days); entry to first service (days); number of live-born pigs/farrowing; % stillbirths; % mummies; % sow mortality; replacement rate (%); culling rate (%); and litters/mated female/year. Differences between cohorts were tested for significance by *t*-test.

### 2.4. Calculation of PRRSV Incidence Risk during Disease Year 3 and over Disease Years 1–3

As in disease years 1 and 2, PRRSV incidence risk in disease year 3 (1 July 2023 to 30 June 2024) was based on the number of new viral entries to a fully managed breeding herd divided by the number of herds at risk in each disease year, according to calculations previously published by the University of Minnesota Dr. Bob Morrison’s Swine Health Monitoring Project (MSHMP) [3,9]. As published, a new introduction was defined either as the entry of PRRSV to a historically naïve breeding herd, or the entry of a PRRSV variant to a historically infected herd with the new variant being ≥2% heterologous to the existing variant(s) based on nucleic acid sequencing of open reading frame 5 [3,10]. In addition, the animal inventories of participating breeding herds in disease year 3 were analyzed using descriptive statistics (mean, median, maximum, minimum) and the differences in proportions of new viral entries/number of participating herds in disease year 3 were calculated and compared to years 1 and 2 using Chi square. The association of disease burden (PRRSV incidence risk) and biosecurity level (NGB COMPLETE vs. NGB INCOMPLETE) over the cumulative three-year period was tested by Chi square. Finally, the PRRSV incidence risk over the cumulative three-year period was calculated using a weighted average to account for differences in the number of participating breeding herds each year.

### 2.5. Re-Assessment of Neighboring Swine Herd Density during Disease Year 3

As published in disease years 1 and 2 [3], calculation of neighboring swine density in disease year 3, defined as 1 July 2023 to 30 June 2024, was repeated by identifying swine herds within an 8.3 km radius of each breeding herd using geographical software (Google Earth, version 7.3.2.5776 and Google Map Developers, Chicago, IL, USA (mapdevelopers.com)). This was important to be certain that area density did not influence the results. The distance of 8.4 km was selected based on published data regarding the ability of PRRSV to be transported via aerosols out to and beyond this distance [7]. In conjunction with mapping, site inspections by approved and trained Pipestone personnel were conducted around each participating breeding herd to validate whether the sites did or did not house pigs, and whether each site was owned and/or managed by Pipestone. This practice had been in place across the Pipestone System for more than 13 years. The evaluation of area density around these herds had been calculated twice a year, including regular updates and site inspections during the third year of the study. The difference in the mean number of neighboring swine herds within an 8.3 km radius of NGB COMPLETE herds and NGB INCOMPLETE herds was analyzed by the Mann–Whitney *U* test (Statistics Kingdom, Melbourne, Australia) (https://www.statskingdom.com/170median_mann_whitney.html, 27 July 2023), with the level of significance set at ≤0.05. Graphs were constructed using Microsoft Excel (Microsoft Corporation, Redmund City, CA, USA) as published, in accordance with the format used by MNSHMP [9].

## 3. Results

### 3.1. Statistical Analysis of KPIs during Disease Years 1 and 2 across Cohorts

Data from the KPIs between the breeding herd cohorts during disease years 1 and 2 are summarized in Table 3. A total of 62 herds qualified for the comparison based on the previously described criteria, and 43 herds (208,918 sows) were categorized as NGB COMPLETE and 19 herds (64,119 sows) as NGB INCOMPLETE. Table 4 summarizes the statistical differences in the means of the 20 KPIs between the two cohorts as determined by *t*- test, with significant differences highlighted in red. Significant differences favoring NGB COMPLETE herds included an increase in total born per farrow (*p* = 0.047), and pigs weaned per farrow (*p* = 0.021), a decrease in pre-weaning mortality rate (*p* = 0.013), and a reduced wean to 1st service interval (*p* = 0.007). While not statistically significant (*p* = 0.15), there was a 0.91 increase in the number of pigs weaned/sow/year in NGB COMPLETE herds versus INCOMPLETE herds. There were also no statistically significant differences in % sow mortality (*p* = 0.18), % stillborn (*p* = 0.77) and % mummies (*p* = 0.17) between cohorts, parameters historically associated with PRRSV infection.

### 3.2. PRRSV Incidence Risk Calculations during Disease Year 3 and over the Cumulative 3-Year Period

**Figure 1:**
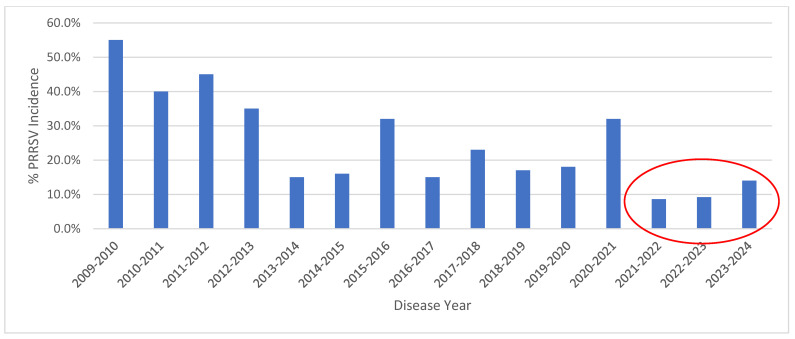
Trend in mean PRRSV incidence risk from disease year 2009–2010 through 2023–2024. Note reduction in PRRSV incidence risk beginning in disease year 2021–2022 through 2023–2024 (circled in red) following the application of Next Generation Biosecurity in July 2021.

During disease year 3 (1 July 2023 to 30 June 2024), a total of 75 breeding herds, consisting of 384,207 sows, were involved in the project. The mean sow inventory was 5123 (min = 1362, max = 12,064) and the median herd size was 5516. The 95% CI from the mean ranged from 4622 to 5624 with a standard deviation of 2100 (Table 2). Across these herds, 11 new PRRSV introductions were recorded for a PRRSV incidence risk of 14.6%. The degree of heterology at ORF 5 across these viruses ranged from 10 to 12%. Of these 11 infected herds, six were categorized as NGB COMPLETE and five as NGB INCOMPLETE. The proportion of positive herds in disease year 3 (11 new viral entries/75 herds, 14.6%) was not significantly different (*p* = 0.77) as compared to disease year 1 (6/69 herds, 8.6% PRRSV incidence risk) or disease year 2 (7/76 herds, 9.2% PRRSV incidence risk) (Table 5). In disease year 3, 58 of the 75 herds (77.3%) were categorized as NGB COMPLETE while 17 (22.7%) were categorized as NGB INCOMPLETE. Across the cumulative 3-year period, the association of PRRSV incidence risk and level of biosecurity favored NGB COMPLETE herds (*p* < 0.0001) and the PRRSV incidence risk over the cumulative 3-year period across all herds was 8.0%. Figure 1 depicts the change in PRRSV incidence risk in the Pipestone System from disease year 2009–2010 through 2023–2024, denoting the system-wide application of NGB from July 2021 to June 2024.

### 3.3. Re-Assessment of Neighboring Swine Herd Density during Disease Year 3

During disease year 3, it was confirmed that no changes had occurred in area herd density as compared to data previously published [3]. Therefore, the median number of neighboring swine herds within an 8.3 km radius of NGB COMPLETE and NGB INCOMPLETE breeding herds were once again determined to be 2.0 and 2.0, respectively.

## 4. Discussion

Throughout global agriculture, herds and flocks are constantly dealing with the relentless pressure of viral diseases, be it from transboundary agents such as the African swine fever virus and highly pathogenic avian influenza virus, or from domestic pathogens such as PRRSV. In the absence of vaccines capable of inducing sterilizing immunity, the key to controlling these pathogens is to prevent introduction to susceptible populations, which results in improved health, welfare, and productivity [11,12]. Recently, in the US, there has been a significant effort towards reducing the impact of PRRSV through the application of Next Generation Biosecurity, and the initial published results are encouraging [3]. In support of this publication, this follow-up communication reports new information consisting of PRRSV incidence risk data from disease year 3, a cumulative incidence risk over disease years 1–3, the association of biosecurity level and PRRSV incidence over the cumulative 3-year period, and describes statistically significant differences in KPIs across the two cohorts during disease years 1 and 2.

As mentioned, KPIs derived from Porcitec performance analyses were used to test differences in breeding herd productivity between NGB COMPLETE and NGB INCOMPLETE cohorts. For this assessment, we selected 62 herds from a well-controlled and finely managed system of pig production. Results of this analysis indicated a statistically significant advantage to NGB COMPLETE herds regarding total born/female, pigs weaned/female, pre-weaning mortality, and wean to 1st service interval. As we attempted to control variability, it was good to see that several critical KPIs that could have biased the outcomes were similar between cohorts, including average parity farrowed (NBG COMPLETE = 3.52 vs. NGB INCOMPLETE = 3.55) and litters/mated female/year (NGB COMPLETE = 2.34 vs. NGB INCOMPLETE = 2.35). It was also good to see that the percent sow mortality was statistically insignificant (*p* = 0.18) between the cohorts, which suggests a high degree of animal care and welfare as it pertains to managing gestating and lactating sows. Possibly most impactful, despite its lack of statistical significance, was the increase of 0.91 pigs weaned/mated female/year, which resulted in 190,115 additional weaned pigs across the 208,918 sows in the 43 NGB COMPLETE herds, clearly a “biologically significant” result. These are interesting outcomes, and one conclusion that could be drawn was that the improvements in productivity observed in the NGB COMPLETE cohort may have been due to reduced PRRSV incidence risk secondary to improvements in biosecurity, supported by the significant association (*p* < 0.0001) between PRRSV incidence in NGB COMPLETE herds vs. NGB INCOMPLETE herds over the 3-year study period. However, it must be disclosed that a major limitation of using the Porcitec program was that the performance analysis did not include an abortion rate and clustered the number of weak born piglets in the pre-weaning mortality metric; therefore, these parameters, known to be characteristic of PRRSV infections in sows, could not be analyzed.

As with all studies, there were strengths and limitations that need to be reported and discussed. One strength of this communication is the generation of additional data across more sows, more herds, and more time, thereby building on the outcomes from disease years 1 and 2. Another strength is the transparency of the reporting. Specifically, while the proportion of positive herds across the three years was not statistically different across the 3-year period, it is important to disclose that the number of new viral introductions in year 3 was numerically greater than the two previous years. Upon investigation, it was observed that of the 11 new infections in disease year 3, four occurred in NGB INCOMPLETE herds which had also been infected in year 2, which is likely due to the lack of an air filtration system. This is a very important point to raise, as the presence or absence of an air filtration system was technically the only difference between the NGB COMPLETE and INCOMPLETE cohorts. Therefore, one conclusion that could be drawn was that the presence of an air filtration system played a role in reducing PRRSV incidence risk as previously reported by Havas and others [12]. In addition, two other herds, one categorized as NGB COMPLETE and another as NGB INCOMPLETE, became infected during herd repopulation, a very chaotic event when the frequency of personnel entry, and pig transport into a farm, are higher than normal, all which could have enhanced viral entry via an aerosol and/or fomite-based route. Finally, another NGB COMPLETE herd became infected shortly after a tornado caused significant damage to the roof of the farrowing facility, resulting in extensive air entry and possible viral entry.

We must also acknowledge that a challenge to the practice of biosecurity is the “human factor”, and NGB is no exception to this rule [13]. The practice of NGB, while very effective, places employees under tremendous pressure and, while it was not quantified, it is possible that “biosecurity fatigue” may have occurred in disease year 3. Alternatively, the high degree of success that occurred in disease years 1 and 2 may have promoted an attitude of complacency, and people may have reduced focus. While we acknowledge the inability to completely control the “human factor”, the fact that all farm personnel were employees of Pipestone and had participated in standardized training and auditing programs controlled this variable to the best of our knowledge and ability.

From the viral perspective, another limitation of NGB could be that certain routes of PRRSV transmission have not yet been identified and therefore, all the necessary protocols may not be in place. In addition, recently evolved variants such as PRRSV 144 L1C appear to be more infectious and contagious than historic variants, which could challenge the efficacy of the biosecurity protocols that were used [14]. As the 144 L1C variant was identified in several of the outbreaks over the three years, viral adaptation could have played a role. Finally, as this study only involved one pig production company, we must acknowledge that NGB may not be practical in other companies.

Clearly, the results from this paper clearly indicate that the NGB approach has not yet been perfected and additional work is needed. However, despite the acknowledged limitations, the ability to report a PRRSV incidence risk of 8.0% over a 3-year period across a pig production system of this size is not only a novel observation, but also low enough that the occasional new infection no longer compromises overall system operations. For the record, it needs to be reported that across all participating herds there was no application of bio-containment strategies. Therefore, the next steps include calculating the cost: the benefit of NGB, evaluating its ability to manage the wean-to-market aspect of biosecurity, and determining its ability to reduce incidence risk of other swine viral diseases. Along these lines, it is worth mentioning that only one of the 75 breeding herds (1.3%) became infected with the porcine epidemic diarrhea virus in disease year 3 (Brands, L., Pipestone Veterinary Services, Pipestone, MN, USA, Personal communication, 1 July 2024). With this in mind, it needs to be acknowledged that the improvements in KPIs noted in the study may have been due in part to the control of other pathogens, not only to improvements in PRRSV incidence risk. This is a very exciting hypothesis and future studies should investigate the effect of NGB on bacterial pathogens, such as *Streptococcus suis* and viral pathogens such as influenza virus A of swine. In addition, should the concept of NGB be applied across species groups, i.e., poultry or dairy cattle, it may help prevent the spread of significant pathogens that plague both livestock sectors, such as the highly pathogenic avian influenza virus.

In closing, this communication supports the previously published conclusion that Next Generation Biosecurity, while not perfect, helps to improve both animal health and productivity through the sustained reduction of PRRSV incidence risk at the level of the breeding herd [3]. This is further evidence that PRRSV can be successfully controlled through improvements in biosecurity. Therefore, the authors hope that this new way of thinking will benefit global animal agriculture, as practitioners and scientists collectively strive to manage the challenge of viral diseases of herds and flocks.

## 5. Conclusions

This communication provides further evidence that improved biosecurity can successfully reduce the impact of PRRS, one of the most economically significant diseases of global agriculture, as well as improve breeding herd productivity. The information from this paper, in combination with its predecessor [3], provides hope and guidance for farmers and veterinarians regarding the management of PRRSV, as well as other domestic and transboundary diseases across herds and flocks throughout the global livestock industry.

## Figures and Tables

**Table 1:** Annual Pipestone PRRSV Incidence Risk 1 July 2009 to 30 June 2021.

Disease Year	% PRRSV Incidence Risk
2009–2010	55%
2010–2011	40%
2011–2012	45%
2012–2013	35%
2013–2014	15%
2014–2015	16%
2015–2016	32%
2016–2017	15%
2017–2018	23%
2018–2019	17%
2019–2020	18%
2020–2021	32%

**Table 2:** Descriptive statistics of breeding herd inventories in disease year 3 across the total number of participating herds in the company and the number of herds in the two cohorts, NGB COMPLETE and NGB INCOMPLETE.

	TOTAL	NGB COMPLETE	NGB INCOMPLETE
# Herds	75	58	17
# Sows	384,207	318,788	65,419
Mean herd size	5123	5590	3473
Median herd size	5516	5708	3183
Maximum herd size	12,064	12,064	6249
Minimum herd size	1362	1397	1362
95% CI	4622–5624	5020–6160	2719–4226
SD	2100	2149	1466

**Table 3:** Descriptive statistics of the inventories from the 62 breeding herds included in the analysis of the key performance indicators from disease years 1 and 2. Values are expressed as number of sows.

Biosecurity Level	# Herds #Sows	Mean Median	Min	Max
NGB complete	43 208,918	4858 5507	930	9907
NGB incomplete	19 64,119	3375 3101	1497	5993

**Table 4:** Statistical analysis of the KPIs across NGB COMPLETE and NGB INCOMPLETE herds.

KPI	NGB COMPLETE	NGB INCOMPLETE	*p*-Value	Difference
Number of farms	43	19	-	+24
Farrowing Rate	88.57	88.79	0.80	−0.22
Total Born Per Farrow	**16.11**	15.81	**0.047**	0.30
Pigs Weaned Per Female	**12.20**	11.77	**0.021**	0.43
Pre-weaning Mortality Rate	**15.85**	17.63	**0.013**	−1.78
Weaned/Mated Female/Year	28.64	27.73	0.15	0.91
% Sow Mortality	11.48	10.71	0.18	0.77
% Repeat Services	3.49	4.18	0.31	0.69
Average Parity Farrowed	3.52	3.55	0.77	0.03
% Multiple Matings	90.17	89.72	0.77	0.46
Wean to 1st Service Interval (days)	**7.13**	8.21	**0.007**	−1.09
% Bred by 5 Days	83.28	80.70	0.09	2.58
Conception Rate (%)	93.68	93.46	0.64	0.22
Age at First Service (days)	212.75	217.72	0.08	−4.97
Entry to 1st Service Interval (days)	9.90	11.30	0.56	−1.40
Liveborn Per Farrow	14.51	14.28	0.13	0.23
% Stillborn	6.51	6.60	0.77	−0.09
% Mummies	3.43	3.07	0.17	0.36
Litters/Mated Female/Year	2.34	2.35	0.70	−0.09
Replacement Rate (%)	59.36	55.40	0.28	3.96
Culling Rate (%)	46.66	42.66	0.11	4.00

**Table 5:** Comparison of the proportion of PRRSV positive breeding herds recorded during disease year 3 as compared to disease years 1 and 2.

	Disease Year 1	Disease Year 2	Disease Year 3
Proportion positive (# new infections/#herds)	8.69% (6/69) ^a^	9.23% (7/76) ^a^	14.6% (11/75) ^a^
# infected herds NGB COMPLETE/total # herds NGB COMPLETE	6.25% (3/48)	3.5% (2/56)	10.3% (6/58)
# herds NGB INCOMPLETE/total # herds NGB INCOMPLETE	14.2% (3/21)	25% (5/20)	29.4% (5/17)

^a^: Differences in the proportions of PRRSV positive herds across the 3 disease years were found to be not significant by Chi square at *p* = 0.77.

## Data Availability

All data are included in the paper.
